# Supplementary material for: Optimal number of spacers in CRISPR arrays
Source: PLoS Comput Biol. 2017 Dec 18;13(12):e1005891. doi: 10.1371/journal.pcbi.1005891 (PMC5749868; doi:10.1371/journal.pcbi.1005891)
Supplement: S1 Appendix — (PDF) [file pcbi.1005891.s001.pdf]

# S1 Appendix

## CRISPR-induced reduction in the viral burst

In the main text we estimated the number of CRISPR spacers that maximizes survival of a host cell. Here are compute the number of spacers which minimizes the viral burst (and thus the number of secondary infections) from a doomed host cell with still functioning CRISPR system. As in Eq. (7), the total interference rate is assumed to be proportional to the total binding probability multiplied by the copy number of viral DNA. This is an overestimating approximation as in reality there is a spreading of a fixed number CRISPR effectors over increasing number of copies of viral DNA, which inevitably makes binding to any given protospacer less probable. Such a reduction in binding efficiency makes survival of viral DNA a “runaway” process: it becomes progressively less plausible to completely exterminate viral DNA after the first round of DNA replication. We also approximate viral DNA replication as a continuous process and obtain the following kinetic equation for the copy number of viral DNA $V(t)$,

$$\frac{dV(t)}{dt} = V(t)\left(D - a\sum_i B_i\right), \tag{S1}$$

with the solution

$$V(t) = \exp\left[\left(D - a\sum_i B_i\right)t\right]. \tag{S2}$$

Here $D$ is the viral duplication rate and it is assumed that initially the host cell contained a single copy of viral DNA, $V(0) = 1$

Without active CRISPR system, the number of viral DNA copies reaches the native burst size $V_b$ after time $\theta$,

$$V_b = \exp\left[D\theta\right]. \tag{S3}$$

Assuming that the viral maturation time $\theta$ is not affected by CRISPR activity, the viral burst in the presence of CRISPR $V_{CRISPR}$ becomes,

$$V_{CRISPR} = V_b \exp\left[-a\sum_i B_i\theta\right] = V_b \exp\left[-\chi\frac{\theta}{\tau}\sum_i B_i\right]. \tag{S4}$$

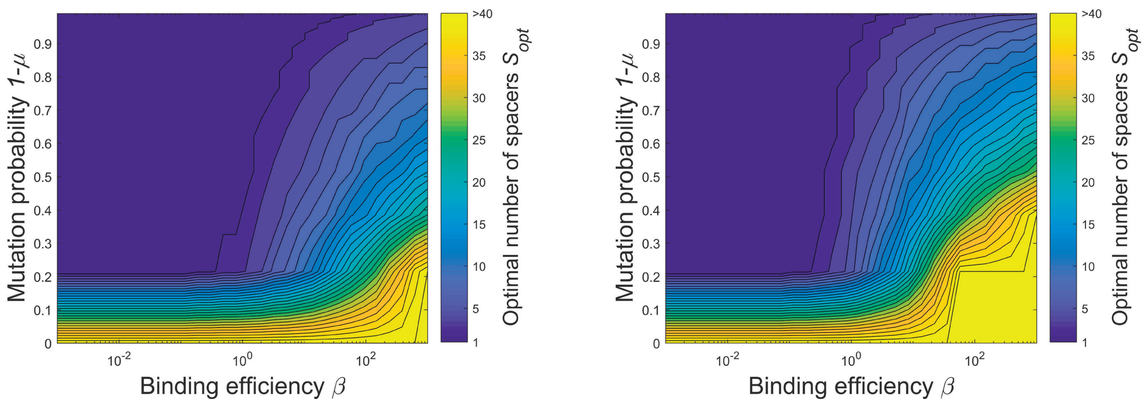


Figure A: **The maximum of the product (S6) for various $\chi$.** The number of spacers $S$ that maximizes expression S6 for: the host cell survival with $\chi = 1.4$ (left panel) and the size of viral burst with $\chi' = \nu\chi = 1.4 * 6.65 \approx 9.3$ (right panel). As in the main text, $m_i = \mu^{i-1/2}$.

The factor $\nu \equiv \theta/\tau$ is the number of cycles of replication of viral DNA and can be estimated from the burst size, $2^\nu = V_b$.

Steps analogous to those leading to Eqs. (13-15) in the main text show that the burst size in host cells infected with viruses with $S$ protospacers each having probability $m_i$ to remain mutation-free is

$$V_{CRISPR} = V_b \prod_{i=1}^{S} \{1 - m_i [1 - \exp(-\nu\chi B_i)]\} \tag{S5}$$

.

Comparing Eq. (S5) to Eq. (15) in the main text reveals that the minimum of the product

$$\prod_{i=1}^{S} \{1 - m_i [1 - \exp(-\nu\chi B_i)]\} \tag{S6}$$

maximizes the host cell survival probability when $\nu = 1$ and minimizes the viral burst size when $\nu$ equals to the number of cycles of replication of viral DNA. For a typical burst size $v_b = 100$, the number of replication cycles $\nu \approx 6.65$ (see Eq. S2 in Appendix S2), which, as seen comparing left and right panels of Fig. A, usually increases the optimal number of spacers (see also Fig. 5(C) in the main text showing the dependence of the optimal number of spacers on $\chi$.)
