# Supplementary material for: Optimal number of spacers in CRISPR arrays
Source: PLoS Comput Biol. 2017 Dec 18;13(12):e1005891. doi: 10.1371/journal.pcbi.1005891 (PMC5749868; doi:10.1371/journal.pcbi.1005891)
Supplement: S2 Appendix — (PDF) [file pcbi.1005891.s002.pdf]

S2 Appendix

Calculation of interference efficiency from experimental data

We use the data from [1], which quantitatively assesses the efficience of interference of a single-spacer CRISPR system against the T7 phage with a perfectly matching protospacer. The DNA abundance from the protospacer region, which is cut by CRISPR effectors, and the reference unaffected by CRISPR are compared to each other. Since the probability for the viral DNA to survive a duplication cycle is $1-I$ (see Eqs. (8,9) in the main text), the number of copies of the protospacer region of viral DNA V_{CRISPR} after ν rounds of duplication is

$$V_{CRISPR} = [2 * (1 - I)]^{\nu}. \tag{S1}$$

The CRISPR-free viral burst size and, presumably, the number of copies of reference regions of phage DNA is $V_b \approx 100$ viruses, thus the average number of virus duplications ν is given by

$$2^{\nu} = V_b = 100,\ \nu = \frac{\ln 100}{\ln 2} \approx 6.65. \tag{S2}$$

The ratio between the amount of DNA from the reference and protospacer regions was reported in [1] to be approximately 100,

$$\frac{V_b}{V_{CRISPR}} \approx 100. \tag{S3}$$

Thus

$$[2 * (1 - I)]^{r} \approx 1,\ I \approx 0.5. \tag{S4}$$

The relation between β and χ that reproduces the interference probability of the single-spacer array from [1] is obtained by inverting the Eq. (9) from the main text and limiting the sum to the first term,

$$\chi = -\ln(1 - I)(1 + 1/\beta) = (1 + 1/\beta)\ln(2). \tag{S5}$$

References

[1] Strotskaya A, Savitskaya E, Metlitskaya A, Morozova N, Datsenko KA, Semenova E, et al. The action of Escherichia coli CRISPRCas system on lytic bacteriophages with different lifestyles and development strategies. Nucleic Acids Research. 2017;(15):gkx042. doi:10.1093/nar/gkx042.

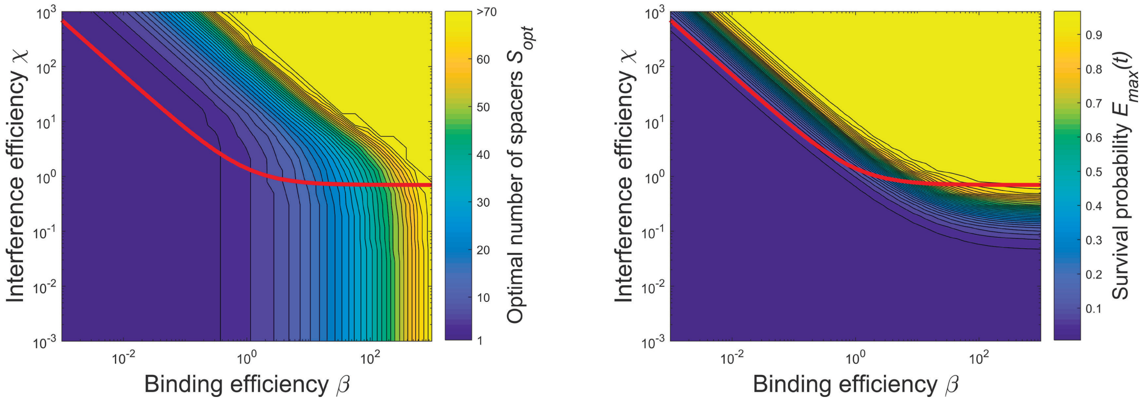

Figure B: **The effects of binding efficiency β and interference efficiency χ on CRISPR performance** The optimal number of spacers (left panel) and the corresponding survival probability (right panel) are shown for various β and χ. The probability for the protospacer to remain mutation-free is $\mu = 0.9$ in both plots. Red line corresponds to the values of β and χ satisfying the restriction $I = 0.5$ given by Eq. S5.
